# Rates of knee arthroplasty in anterior cruciate ligament reconstructed patients: a longitudinal cohort study of 111,212 procedures over 20 years

**DOI:** 10.1080/17453674.2019.1639360

**Published:** 2019-07-10

**Authors:** Simon G F Abram, Andrew Judge, Tanvir Khan, David J Beard, Andrew J Price

**Affiliations:** a Nuffield Department of Orthopaedics, Rheumatology and Musculoskeletal Sciences, University of Oxford, UK;; b NIHR Biomedical Research Centre, Oxford;; c Musculoskeletal Research Unit, University of Bristol;; d NIHR Biomedical Research Centre, Bristol;; e Faculty of Medicine & Health Sciences, University of Nottingham, UK

## Abstract

Background and purpose — Long-term rates of knee arthroplasty in patients with anterior cruciate ligament (ACL) injury who undergo ligament reconstruction (ACLr) are unclear. We determined this risk of arthroplasty through comparison with the general population.

Patients and methods — All patients undergoing an ACLr in England, 1997–2017, were identified from national hospital statistics. Patients subsequently undergoing a knee arthroplasty were identified and survival analysis was performed (survival without undergoing knee arthroplasty). A Cox proportional hazards model was used to identify factors associated with knee arthroplasty. Relative risk of knee arthroplasty (total or partial) in comparison with the general population was determined.

Results — 111,212 ACLr patients were eligible for analysis (mean age 29; 77% male). Overall, 0.46% (95% confidence interval [CI] 0.40–0.52) ACLr patients underwent knee arthroplasty within 5 years, 0.97% (CI 0.82–1.2) within 10 years, and 1.8% (CI 1.4–2.3) within 15 years. Knee arthroplasty risk was greater in older age groups and women. In comparison with the general population, the relative risk of undergoing arthroplasty at a younger age (at time of arthroplasty) was elevated: at 30–39 years (risk ratio [RR] 20; CI 11–35), 40–49 years (RR 7.5; CI 5.5–10), and 50–59 years (RR 2.5; CI 1.8–3.5), but not 60–69 years (RR 1.7; CI 0.93–3.2).

Interpretation — Patients sustaining an ACL injury who undergo ACLr are at elevated risk of subsequent knee arthroplasty in comparison with the general population. Although the absolute rate of arthroplasty is low, the risk of arthroplasty at a younger age is particularly elevated. When the outcome of shared decision-making is ACLr, this data will help inform patients and clinicians about the long-term risk of requiring knee arthroplasty.

Approximately 25–50% of patients with anterior cruciate ligament (ACL) injuries subsequently undergo ACL reconstruction surgery (Frobell et al. 2010, Collins et al. 2013, Nordenvall et al. 2014). This corresponds to a rate of ACL reconstruction thst has been reported at approximately 45/100,000 population per year in the United States and 24/100,000 in the United Kingdom (Buller et al. 2015, Abram et al. 2019). ACL reconstruction may either be performed early to stabilize the knee and prevent a further pivoting injury or may be delayed and performed only in patients with knee instability despite physiotherapy (Frobell et al. 2010).

A key concern is that up to half of patients with a history of ACL injury develop signs of radiographic osteoarthritis within 10–15 years (Lohmander et al. 2007, Ajuied et al. 2014). ACL reconstruction and rehabilitation aims to stabilize the knee to reduce the risk of further injury with additional damage to the chondral surfaces and menisci (Kay et al. 2018, Mok et al. 2019). Many surgeons believe that ACL reconstruction is protective against osteoarthritis for the ACL-injured patient, notwithstanding the impact from the original injurious episode (Marx et al. 2003). Indeed, there is some reported evidence from a cohort of young, active individuals that ACL reconstruction reduces the risk of further meniscal and cartilage damage, in comparison with nonoperative management (Dunn et al. 2004). A previous meta-analysis (Ajuied et al. 2014) indicated that radiographic osteoarthritis may develop less frequently in ACL-injured knees managed with reconstruction in comparison with nonoperative treatment, but results are conflicting, with 1 large cohort finding no such association (Nordenvall et al. 2014). The use of radiological grading as an outcome or indicator of osteoarthritis is relatively subjective and may not reflect a patient’s symptoms (Parry et al. 2017).

Knee arthroplasty is a powerful and obvious surrogate marker for severe osteoarthritis, combining the severity of symptoms with radiological assessment (Bruyere et al. 2008, Raynauld et al. 2011, Carr et al. 2012). Population intervention rates for ACL reconstruction and knee arthroplasty have increased over the last 20 years in England but association between these interventions is unknown (Abram et al. 2019). Studies utilizing knee arthroplasty rates are scarce; however, case-control studies have suggested that ACL injury may be associated with up to a 7-times greater odds of knee arthroplasty (Leroux et al. 2014, Khan et al. 2018). Due to the limitations of these case-control studies, we determined the long-term risk (up to 15 years) of knee arthroplasty in patients with a history of ACL injury and undergoing surgical reconstruction, from an analysis of 20 years of longitudinal data from the complete National Health Service database for England, UK. 

## Patients and methods

### Study design, setting, and data sources

We performed a longitudinal cohort study utilizing the national healthcare records for England, UK. National Hospital Episode Statistics (HES) data were acquired for the purposes of this study for the period between April 1, 1997 and March 31, 2017 (NHS Digital; application reference: DARS-NIC-68703). HES includes all National Health Service (NHS) care episodes, whether delivered in NHS hospitals or independent treatment centers, and also privately funded patients treated within NHS England hospitals. Surgical procedures, primary and secondary diagnoses, demographic and geographic data are recorded. Mortality data from the Office for National Statistics (ONS) mortality dataset (all in-hospital or community deaths) was used to adjust the number at risk when performing survival analysis (see below).

### Procedures and participants

Patient HES records were identified from Classification of Surgical Operations and Procedures (OPCS-4) codes associated with inpatient care episodes for ACL reconstruction (see Supplementary data: Appendix 1). The first ACL reconstruction per patient was included, contralateral or revision procedures were not included, and cases undergoing multi-ligament reconstruction, ACL repair, or synthetic ligament surgery were excluded.

### Controls

An arthroplasty control population was extracted for the year 2016–17: all patients undergoing arthroplasty in this year without a history of ACL reconstruction in previous years (records of ACL injury specifically were not available). This control population was analyzed only in the secondary outcome analysis of relative risk of knee arthroplasty for the ACL reconstruction population in comparison with this population. The population rate of arthroplasty for the control population was determined from the number of arthroplasties performed per age group population using mid-year population estimates available from the ONS.

### Outcomes

The primary outcome was the rate of knee arthroplasty following ACL reconstruction. All HES and ONS records (including prior and subsequent hospital admission records) were then extracted for each patient with OPCS-4 codes for either ACL reconstruction or knee arthroplasty. Laterality coding was available to enable matching of procedures both by patient and by knee side (left vs. right). For patients undergoing ACL reconstruction, concurrent chondral or meniscal procedure codes were identified and included for comparison with isolated ACL reconstruction procedures. Per patient, only the first (primary) ACL reconstruction was included. Patients undergoing simultaneous knee arthroplasty and ACL reconstruction in the same hospital episode were excluded as these were not considered to be relevant to this study. Secondary outcomes were the relative odds of knee arthroplasty by a range of patient factors (defined below), and the relative risk of knee arthroplasty versus the control population defined above.

### Confounders

The following potential confounding variables were analyzed: time (year of treatment), sex, age group, year of treatment, Charlson comorbidity index (derived with maximum 5-year diagnosis code lookback period), index of multiple deprivation (quintile derived from regional factors in England including average income, employment, education, housing, and crime; 1 = least deprived area, 5 = most deprived), rurality, ethnicity, and any concurrent chondral or meniscal surgery. These variables were selected a priori due to their potential importance in determining outcome, treatment choices or eligibility, or access to healthcare.

### Statistics

In accordance with ONS and NHS Digital guidance, small numbers were suppressed where required. The rates of knee arthroplasty following ACL reconstruction at 5, 10, and 15 years were calculated as the absolute proportion and reported with the 95% confidence interval (CI) for the population sample size.

A Cox proportional hazards model was used to calculate hazard ratios for subsequent arthroplasty over time, stratified by the confounders described in the previous section. Unadjusted and covariate-adjusted hazard ratios were calculated. Cases missing essential data (age, sex, procedure date, procedure laterality) were excluded from the study during data cleaning. Cases missing nonessential data (index of multiple deprivation, ethnicity, rurality) were included except for analyses adjusting for these specific variables.

Risk over time was also analyzed with Kaplan–Meier survival analysis to estimate and graphically report the long-term risk of undergoing knee arthroplasty (total or partial) using the full cohort data up to 20 years following ACL reconstruction: overall and stratified by patient age at the time of reconstruction and by sex.

To determine the relative risk of knee arthroplasty after ACL reconstruction in comparison with the general population, the absolute rate of knee arthroplasty in 2016–17 was calculated for patients with and without a history of previous ACL reconstruction (records of ACL injury specifically were not available in this database). For the ACL population, the numerator was the number of matched, same side, knee arthroplasty procedures. For the general population, the numerator was all other knee arthroplasty patients without a history of ACL reconstruction. The denominator for the ACL population was all living patients in 2016–17 without a prior history of knee arthroplasty in the index knee. For the general population, denominator data were extracted from the ONS national population estimates. The relative annual risk (risk ratio) of knee arthroplasty for these respective populations within the most recent years of data (2016–17) was then calculated. To aid interpretation and for clinical relevance, both the absolute and relative risk estimates were stratified according to the age of the patient in 2016–17, irrespective of the year of previous ACL reconstruction, where applicable.

Stata v15.1 (StataCorp, College Station, TX, USA) was used to perform all analyses. Confidence intervals are reported at the 95% level.

### Ethics, funding, and potential conflicts of interest

The study was approved by the NHS Digital Independent Group Advising on the Release of Data (IGARD) committee (application reference: DARS-NIC-68703). No other ethical approval was required.

This report is independent research supported by the National Institute for Health Research (NIHR), Oxford Biomedical Research Centre (BRC), and Bristol Biomedical Research Centre (BRC). The views expressed in this publication are those of the authors and not necessarily those of the NHS, the National Institute for Health Research or the Department of Health and Social Care.

All authors have completed the Unified Competing Interest form (available on request from the corresponding author). AJ has received consultancy fees from Freshfields Bruckhaus Deringer (on behalf of Smith & Nephew Orthopaedics Limited), and is a member of the Data Safety and Monitoring Board (which involved receipt of fees) from Anthera Pharmaceuticals, Inc. All other authors declare no conflicts of interest.

## Results

**Figure 1.**
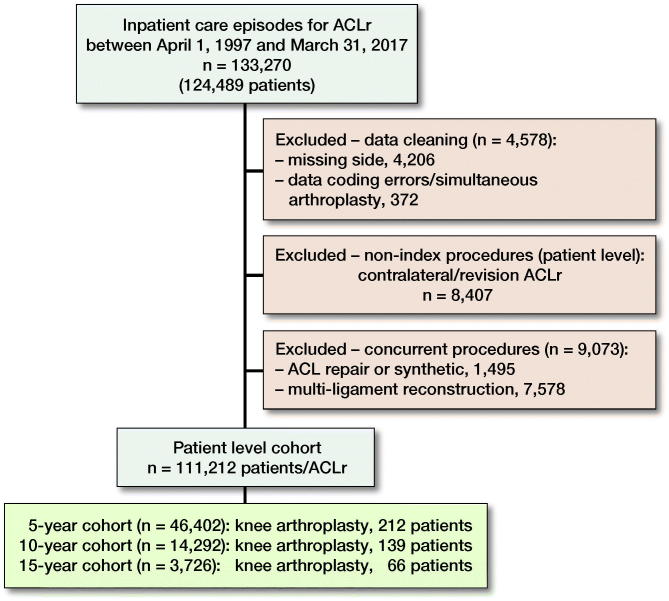
Flow chart illustrating extraction of patient level cohort.

Between April 1, 1997 and March 31, 2017, 124,489 patients underwent ACL surgery, of which 111,212 patients were included in the analysis, with a mean age of 29 (SD 10) (Figure 1) and mean follow up 5.9 years (range 0–20 years; SD 4.2) during which time 0.54% (600/111,212; CI 0.50–0.58) underwent subsequent arthroplasty. There was a greater proportion of male patients (77%) and the most common age group was 20–29 years (43%) (Table 1, see Supplementary data).

**Table 2.** Hazard ratios (subsequent TKA within maximum of 20 years) of ACL reconstruction

Factor	Risk of subsequent TKA	
	Unadjusted HR (CI)	Adjusted HR (CI)
Sex		
Male	1.0	1.0
Female	2.4 (2.1–2.9)	1.5 (1.3–1.8)
Age (years)		
< 20	–	–
20–29	1.0	1.0
30–39	6.2 (4.4–8.8)	6.2 (4.4–8.8)
40–49	20 (14–28)	19 (13–26)
50–59	46 (32–66)	42 (29–61)
≥ 60	–	–
Year of ACL reconstruction		
per year	1.0 (1.0–1.1)	1.0 (0.99–1.0)
Charlson comorbidity index		
per unit	1.1 (1.0–1.1)	1.0 (0.96–1.1)
Index of multiple deprivation (quintile)		
1 = least	1.0	1.0
2	1.1 (0.9–1.5)	1.2 (0.90–1.5)
3	1.1 (0.9–1.5)	1.2 (0.94–1.6)
4	1.2 (0.95–1.6)	1.6 (1.2–2.1)
5 = most	1.4 (1.1–1.8)	2.0 (1.5–2.6)
Rurality		
Urban	1.0	1.0
Rural	1.2 (0.94–1.4)	1.1 (0.86–1.3)
Ethnicity		
White	1.0	1.0
Asian	0.35 (0.18–0.67)	0.40 (0.21–0.77)
Black	–	–
Mixed	–	–
Other	–	–
Concurrent:		
Isolated ACLr	1.0	1.0
Chondral surgery	2.0 (1.3–3.2)	1.5 (0.92–2.3)
Meniscal surgery^a^	0.39 (0.29–0.53)	0.41 (0.30–0.56)

TKA = total or partial knee arthroplasty.

HR = hazard ratio; CI = 95% confidence interval.

a with or without concurrent chondral surgery.

**Figure 2.**
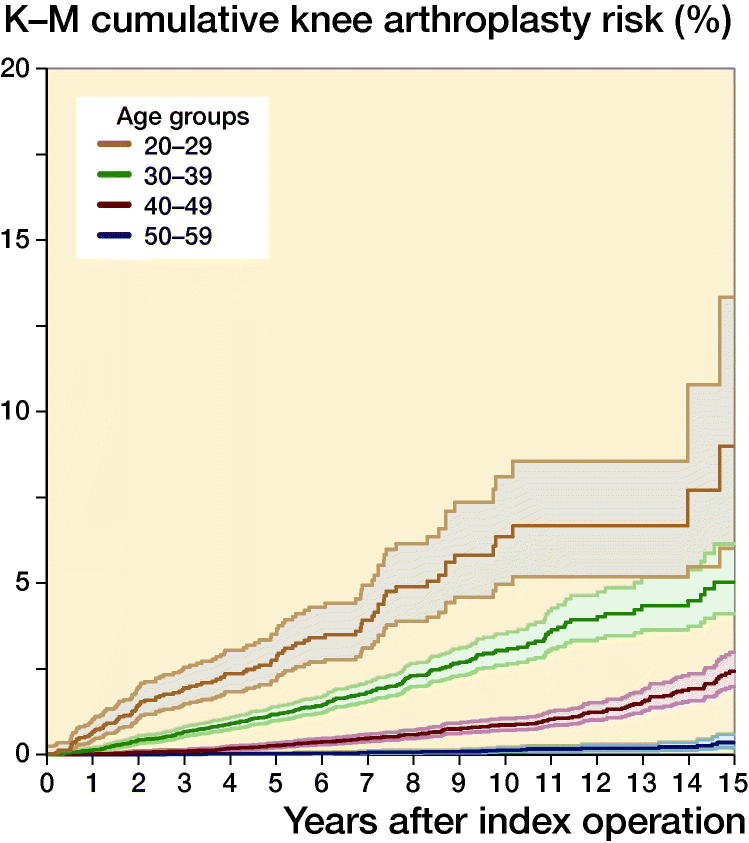
Kaplan–Meier cumulative risk of undergoing knee arthroplasty following ACL reconstruction by age group. Age group < 20 years and ≥ 60 suppressed due to small numbers; shaded areas represent 95% confidence intervals.

**Figure 3.**
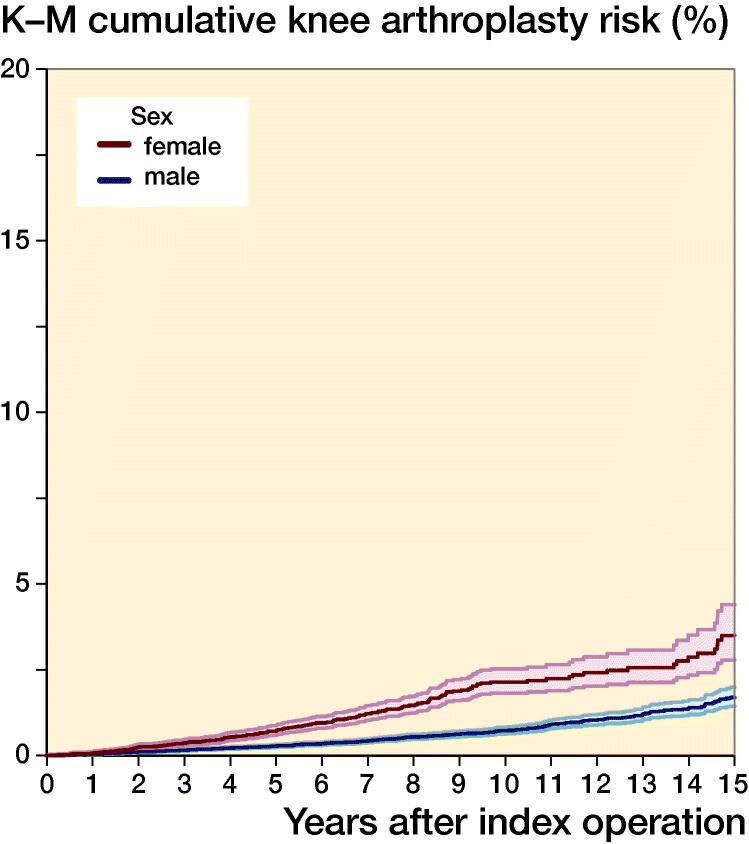
Kaplan–Meier cumulative risk of undergoing knee arthroplasty following ACL reconstruction by sex. Age group < 20 years and ≥ 60 suppressed due to small numbers; shaded areas represent 95% confidence intervals.

Overall, 0.46% (212/46,402; CI 0.40–0.52) ACL reconstruction patients underwent knee arthroplasty within 5 years, 0.97% (139/14,292; CI 0.82–1.2) within 10 years, and 1.8% (66/3,726; CI 1.4–2.3) within 15 years (Table 1, see Supplementary data). The risk was greatly elevated in patients who were older at the time of their index ACL reconstruction (Table 2, Figure 2). In comparison with patients aged 20–29, patients aged 30–39 at the time of their ACL reconstruction were 6.2 times (CI 4.4–8.8), and patients 40–49 were 19 times (CI 13–26), more likely to undergo subsequent knee arthroplasty. The risk of subsequent knee arthroplasty was also elevated in female patients (Figure 3) and patients from the most deprived regions, but lower in patients of Asian ethnicity in comparison with White ethnicity (Table 2). In comparison with isolated ACL reconstruction, concurrent chondral surgery did not significantly alter the rate of subsequent arthroplasty (adjusted HR 1.5; CI 0.92–2.3), but the rate of subsequent arthroplasty was lower in patients with a record of concurrent meniscal surgery (adjusted HR 0.41; CI 0.30–0.56) (Table 2).

The absolute risk of undergoing knee arthroplasty with a history of ACL reconstruction versus without a history of ACL reconstruction is summarized in Table 3. The absolute risks in the ACL reconstruction cohort were low, ranging from 0.04% (CI 0.02–0.06) per year between the ages of 30 and 39 years, to 0.72% (CI 0.34–1.3) per year between the ages of 60 and 69 years. Relative to the general population of patients without a history of ACL reconstruction, patients with a history of ACL reconstruction were considerably more likely to subsequently undergo knee arthroplasty at a younger age, at 30–39 years (risk ratio [RR] 20; CI 11–35), 40–49 years (RR 7.5; CI 5.5–10), and 50–59 years (RR 2.5; CI 1.8–3.5), but not 60–69 years (RR 1.7; CI 0.9–3.2). Overall, for all patients (30–69 years), the relative risk was not significantly elevated (RR 1.1; CI 0.9–1.4) (Table 3). 

**Table 3.** Rates and relative risk of undergoing TKA by age at TKA in 2016 (with versus without a history of ACL reconstruction)

Age at TKA	Prior ACLr Annual rate TKA/10^5^ (CI)	Without prior ACLr Annual rate TKA/10^5^ (CI)	Relative risk RR (CI)
30–39, n	37 (20–63)	1.9 (1.6–2.2)	20 (11–35)
%	0.04 (0.02–0.06)	0.00 (0.00–0.00)	
40–49, n	186 (134–252)	25 (24–26)	7.5 (5.5–10)
%	0.19 (0.13–0.25)	0.02 (0.02–0.03)	
50–59, n	384 (269–531)	151 (148–153)	2.5 (1.8–3.5)
%	0.38 (0.27–0.53)	0.15 (0.15–0.15)	
60–69, n	717 (345–1,315)	414 (408–419)	1.7 (0.9–3.2)
%	0.72 (0.34–1.32)	0.41 (0.41–0.42)	
Overall, n^a^	148 (120–180)	133 (131–134)	1.1 (0.9–1.4)
%	0.15 (0.12–0.18)	0.13 (0.13–0.13)	

TKA = total or partial knee arthroplasty; ACLr = anterior cruciate ligament reconstruction; CI = 95% confidence interval.

a 30–69 years

## Discussion

### Principal findings

In this nationwide retrospective cohort study, we found that 1.8% of patients undergo knee arthroplasty within 15 years of ACL reconstruction. Annual rates of subsequent knee arthroplasty at a young age were elevated in comparison with the general population, suggesting an association with progressive osteoarthritis. The rate of arthroplasty in patients aged 30–39 years, when undergoing arthroplasty, was 21 times higher than anticipated for the general population but the annual rate of arthroplasty in this age group is still very low at 0.04% per year. Risk ratios were lower in increasing age groups and, overall, there was no significant difference in annual arthroplasty rates when all patients (30–69 years) were analyzed together.

### Comparison with other studies

2 case-control studies have reported rates of knee arthroplasty following ACL “injury” and ACL reconstruction, respectively, at 7 times the odds of the general population (Leroux et al. 2014, Khan et al. 2018). Both studies had a number of limitations related to study design and control group matching. The ACL “injury” study could not identify those patients who had undergone surgical interventions and neither study could identify knee “laterality,” precluding matching of the affected knee and potentially leading to overestimation of arthroplasty rates. Overall (all patients aged 30–69 years), patients with a history of ACL reconstruction did not have a significantly elevated annual risk of arthroplasty in comparison with expected rates for the general population. In comparison, however, the annual rate of arthroplasty was elevated in younger age groups to a maximum of 20 times greater risk of undergoing knee arthroplasty at a young age (30–39 years). These findings suggest that ACL injury is an independent risk factor for early osteoarthritis development and may accelerate progression to severe osteoarthritis in susceptible individuals.

There remains uncertainty over whether ACL reconstruction decreases the risk of osteoarthritis following ACL injury (Ajuied et al. 2014, Nordenvall et al. 2014). ACL reconstruction, once performed, may reduce the risk of further pivoting injuries and associated chondral or meniscal damage (Dunn et al. 2004, Fithian et al. 2005, Lohmander et al. 2007). The timing of ACL reconstruction was unknown in our cohort and, being observational, this study cannot determine at a patient level whether the decision to undergo an ACL reconstruction was protective or not. Considering the comparison of our findings with the higher rate of knee arthroplasty in the ACL injury case-control study, the results cannot be interpreted as a comparison of nonoperative care versus operative care of a ruptured ACL (Khan et al. 2018). The case-control study could not distinguish patients who had undergone ACL reconstruction from those who had not, and there is a considerable risk of confounding by indication—for example, patients with pre-existing osteoarthritis would be unlikely to undergo ACL reconstruction but would still have been included (Frobell et al. 2013, Khan et al. 2018). Given the low absolute rate of arthroplasty, however, a very large sample size would be required to determine the relative risk of arthroplasty after nonoperative versus operative management of ACL injury in a randomized study. This would likely be cost-prohibitive and therefore our findings currently represent the best available evidence in this area.

In revision ACL reconstruction, meniscal and chondral pathology has been shown to be associated with inferior patient-reported outcomes (Webster et al. 2018). However, our study found no association between concurrent chondral surgery and subsequent knee arthroplasty. We observed reduced rates of knee arthroplasty in patients who had undergone simultaneous meniscal surgery (partial meniscectomy or repair). These meniscal procedures were combined due to small numbers of repairs, particularly earlier in the study period, and some dual coding – meaning variation in outcome by the specific type of meniscal procedure could not be determined. The lower rate of arthroplasty in this group was surprising, but similar to the findings following ACL reconstruction with simultaneous meniscal debridement reported in a previous case-control study (Leroux et al. 2014). These findings do, however, conflict with relative risk assumptions based on meniscectomy case series where patients with meniscal injury are known to be at greater risk of radiographic arthritic progression (Suter et al. 2016).

In contrast to a previous study, year of treatment did not influence the rate of arthroplasty, suggesting that changes over time in ACL reconstruction techniques, or greater use of first-line nonoperative management strategies, did not significantly alter the observed rate of arthroplasty at a population level (Leroux et al. 2014). This is an interesting observation as, over the same study time period, the rate of ACL reconstruction being performed in England has risen by 1,100% to current rates of 24/10^5^ population per year (Abram et al. 2019). Therefore, despite a rapid rise in intervention rate, rates of subsequent knee arthroplasty have remained stable for this population. This may suggest that, rather than changing indications for ACL reconstruction over this time, the increased intervention rate could represent a correction from previous national under-provision of the procedure or, alternatively, rising rates of injury. Indeed, the rate of intervention in England remains lower than rates in other countries that are as high as 52/10^5^ in Australia (Janssen et al. 2012).

Female sex was found to be associated with a higher rate of arthroplasty and previously female patients have also been shown to be at higher risk of knee arthroplasty after general knee arthroscopy in data from the United States (Boyd and Gradisar 2016). In addition to age and sex, patient-reported ethnicity also influenced this outcome, with a higher rate of arthroplasty in patients reporting white ethnicity in comparison with Asian ethnicity. The reason for this observation is uncertain but would be in accordance with previous studies indicating differences in healthcare access and care-seeking behavior in association with socioeconomic, cultural, occupational, and psychological factors (Adamson et al. 2003, Judge et al. 2010).

There has been one previous clinical trial investigating the role of nonoperative management for ACL injuries and there is another ongoing clinical management strategy trial in the United Kingdom (Frobell et al. 2010, 2013, Beard et al. 2016). Changing treatment practices have not, so far, altered the observed rate of knee arthroplasty following ACL reconstruction at a population level in England, but these studies may lead to the development of new treatment guidance. Recently, there has been increased scrutiny on the need for individualized patient consent in clinical practice (Chan et al. 2017). For patients that do require ACL reconstruction, our study presents important new evidence to inform patients and clinicians of the risk of later requiring knee arthroplasty following ACL reconstruction, clearly stratified by patient factors including age group and sex.

### Strengths and limitations

This paper comprises the largest cohort of ACL reconstruction patients that has been reported and, using national longitudinal cohort data, we have been able to report precisely the rate of subsequent arthroplasty, stratified by patient-specific risk factors, and have determined the relative risk of arthroplasty in comparison with the general population. An observational cohort study such as this is, however, unable to determine whether ACL reconstruction exerted any protective effect against the development of end-stage osteoarthritis. It is unclear whether the key driver of the risk of osteoarthritis following ACL rupture is the original injury or damage to the knee from subsequent pivoting instability episodes (Dunn et al. 2004, Lohmander et al. 2007, Kay et al. 2018, Mok et al. 2019). Other unmeasured factors that may determine the risk of progression to osteoarthritis include genetic, biochemical, and biomechanical factors (Lohmander et al. 2007). In some circumstances, ACL reconstruction may be performed to stabilize a knee to facilitate a partial or total knee arthroplasty and these individuals are unlikely to be representative of the ACL “injury” population (Krishnan and Randle 2009, Weston-Simons et al. 2012). For this reason, we excluded patients undergoing simultaneous knee arthroplasty and ACL reconstruction. The intra-articular ligament reconstruction codes used to identify ACL reconstruction will also have captured posterior cruciate reconstruction procedures. These procedures could not be separately identified but are very rare in other series, and therefore these procedures are unlikely to have materially altered the findings of this study (Årøen et al. 2013).

Despite data cleaning to exclude patient procedures missing a side or with date coding errors, it is inevitable that some other coding errors will have persisted. For HES data, although the specific coding accuracy of ACL reconstruction and arthroplasty procedures has not been determined, other fields from which the Charlson comorbidity index is derived and records of serious vascular diagnoses have been shown to correlate strongly with primary care records in England (Wright et al. 2012, Crooks et al. 2015).

Radiographic or patient-reported outcome data were not available for analysis. This is an important consideration, but knee arthroplasty does represent an objective marker of severe symptomatic osteoarthritis that is clinically relevant and of high importance for patients (Lohmander et al. 2007). Given the reluctance to perform arthroplasty, especially at a young age, it must be noted, however, that the rate of knee arthroplasty will be lower than the rate of radiographic osteoarthritis and also the overall healthcare burden of symptomatic knee pathology in this population.

## Conclusion

Patients with a history of ACL injury and ACL reconstruction are at an increased risk of subsequent knee arthroplasty, especially at a younger age, in comparison with the general population; however, the absolute rate of arthroplasty is low. The relative risk of knee arthroplasty had these patients been managed nonoperatively is unknown and further work is required to refine treatment recommendations following ACL injury. When ACL reconstruction is undertaken, our work will help to inform patients and clinicians of the risk of the undesirable long-term outcome of knee arthroplasty. 

## Supplementary data

Table 1 and Appendix are available as supplementary data in the online version of this article, http://dx.doi.org/10.1080/ 17453674.2019.1639360

## Supplementary Material

Supplemental Material

## References

[CIT0001] Abram S G F, Price A J, Judge A, Beard D J Anterior cruciate ligament (ACL) reconstruction and meniscal repair rates have both increased in the past 20 years in England: hospital statistics from 1997 to 2017. Br J Sports Med 2019; pii: bjsports-2018-100195. [Epub ahead of print] 10.1136/bjsports-2018-100195 30661013

[CIT0002] Adamson J, Ben-Shlomo Y, Chaturvedi N, Donovan J Ethnicity, socio-economic position and gender: do they affect reported health-care seeking behaviour? Soc Sci Med 2003; 57(5): 895–904. 12850114 10.1016/s0277-9536(02)00458-6

[CIT0003] Ajuied A, Wong F, Smith C, Norris M, Earnshaw P, Back D, Davies A Anterior cruciate ligament injury and radiologic progression of knee osteoarthritis. Am J Sports Med 2014; 42(9): 2242–52. 24214929 10.1177/0363546513508376

[CIT0004] Årøen A, Sivertsen E A, Owesen C, Engebretsen L, Granan L P An isolated rupture of the posterior cruciate ligament results in reduced preoperative knee function in comparison with an anterior cruciate ligament injury. Knee Surgery, Sport Traumatol Arthrosc 21(5): 1017–22. 10.1007/s00167-012-2132-1 22801932

[CIT0005] Beard D J, Cook J, Campbell H, Monk A, Wilson C, O’Leary S, Jackson W, Davies L, Carr A, Price A, Haddad F, Barker K, Lamb S The ACL SNNAP Trial: ACL surgery necessity in non acute patients. NIHR HTA; 2016 Source: https://www.hra.nhs.uk/planning-and-improving-research/application-summaries/research-summaries/the-acl-snnap-trial-acl-surgery-necessity-in-non-acute-patients/

[CIT0006] Boyd J A, Gradisar I M Total knee arthroplasty after knee arthroscopy in patients older than 50 years. Orthopedics 2016; 39(6): 1–4. 10.3928/01477447-20160719-01 27459139

[CIT0007] Bruyere O, Pavelka K, Rovati LC, Gatterová J, Giacovelli G, Olejarová M, Deroisy R, Reginster J Y Total joint replacement after glucosamine sulphate treatment in knee osteoarthritis: results of a mean 8-year observation of patients from two previous 3-year, randomised, placebo-controlled trials. Osteoarthr Cartilage 2008; 16(2): 254–60. 10.1016/j.joca.2007.06.011 17681803

[CIT0008] Buller L T, Best M J, Baraga M G, Kaplan L D Trends in anterior cruciate ligament reconstruction in the United States. Orthop J Sport Med 2015; 3(1): 232596711456366. 10.1177/2325967114563664 PMC4555588 26535368

[CIT0009] Carr A J, Robertsson O, Graves S, Price A J, Arden N K, Judge A, Beard D J Knee replacement. Lancet 2012; 379(9823): 1331–40. 22398175 10.1016/S0140-6736(11)60752-6

[CIT0010] Chan S W, Tulloch E, Cooper E S, Smith A, Wojcik W, Norman J E Montgomery and informed consent: where are we now? BMJ 2017; 2224(May): j2224. 10.1136/bmj.j2224 28500035

[CIT0011] Collins J E, Katz J N, Donnell-Fink L A, Martin S D, Losina E Cumulative incidence of ACL reconstruction after ACL injury in adults. Am J Sports Med 2013; 41(3): 544–9. 23302260 10.1177/0363546512472042 PMC3896975

[CIT0012] Crooks C J, West J, Card T R A comparison of the recording of comorbidity in primary and secondary care by using the Charlson Index to predict short-term and long-term survival in a routine linked data cohort. BMJ Open 2015; 5(6): 1–9. 10.1136/bmjopen-2015-007974 PMC4458584 26048212

[CIT0013] Dunn W R, Lyman S, Lincoln A E, Amoroso P J, Wickiewicz T, Marx R G The effect of anterior cruciate ligament reconstruction on the risk of knee reinjury. Am J Sports Med 2004; 32(8): 1906–14. 15572320 10.1177/0363546504265006

[CIT0014] Fithian D C, Paxton E W, Lou Stone M, Luetzow W F, Csintalan R P, Phelan D, Daniel D M Prospective trial of a treatment algorithm for the management of the anterior cruciate ligament-injured knee. Am J Sports Med 2005; 33(3): 335–46. 15716249 10.1177/0363546504269590

[CIT0015] Frobell R B, Roos E M, Roos H P, Ranstam J, Lohmander L S A randomized trial of treatment for acute anterior cruciate ligament tears. N Engl J Med 2010; 363(4): 331–42. 20660401 10.1056/NEJMoa0907797

[CIT0016] Frobell R B, Roos H P, Roos E M, Roemer F W, Ranstam J, Lohmander L S Treatment for acute anterior cruciate ligament tear: five year outcome of randomised trial. BMJ 2013; 346(jan24_1): f232. 23349407 10.1136/bmj.f232 PMC3553934

[CIT0017] Janssen K W, Orchard J W, Driscoll T R, van Mechelen W High incidence and costs for anterior cruciate ligament reconstructions performed in Australia from 2003–2004 to 2007–2008: time for an anterior cruciate ligament register by Scandinavian model? Scand J Med Sci Sport 2012; 22(4): 495–501. 10.1111/j.1600-0838.2010.01253.x 21210852

[CIT0018] Judge A, Welton N J, Sandhu J, Ben-Shlomo Y Equity in access to total joint replacement of the hip and knee in England: cross sectional study. BMJ 2010; 341: c4092 20702550 10.1136/bmj.c4092 PMC2920379

[CIT0019] Kay J, Memon M, Shah A, Meng Y, Kristian Y, Devin S Earlier anterior cruciate ligament reconstruction is associated with a decreased risk of medial meniscal and articular cartilage damage in children and adolescents: a systematic review and meta- analysis. Knee Surgery, Sport Traumatol Arthrosc 2018; 0(0): 0. 10.1007/s00167-018-5012-5 29876862

[CIT0020] Khan T, Alvand A, Prieto-Alhambra D, Culliford D J, Judge A, Jackson W F, Scammell B E, Arden N K, Price A J ACL and meniscal injuries increase the risk of primary total knee replacement for osteoarthritis: a matched case-control study using the Clinical Practice Research Datalink (CPRD). Br J Sports Med 2018; pii: bjsports-2017-097762. [Epub ahead of print] 10.1136/bjsports-2017-097762 29331994

[CIT0021] Krishnan S R, Randle R ACL reconstruction with unicondylar replacement in knee with functional instability and osteoarthritis. J Orthop Surg Res 2009; 4(1): 1–5. 20017914 10.1186/1749-799X-4-43 PMC2806375

[CIT0022] Leroux T, Ogilvie-Harris D, Dwyer T, Chahal J, Gandhi R, Mahomed N, Wasserstein D The risk of knee arthroplasty following cruciate ligament reconstruction: a population-based matched cohort study. J Bone Joint Surg Am 2014; 96(1): 2–10. 24382718 10.2106/JBJS.M.00393

[CIT0023] Lohmander L S, Englund P M, Dahl L L, Roos E M The long-term consequence of anterior cruciate ligament and meniscus injuries: Osteoarthritis. Am J Sports Med 2007; 35(10): 1756–69. 17761605 10.1177/0363546507307396

[CIT0024] Marx R G, Jones E C, Angel M, Wickiewicz T L, Warren R F Beliefs and attitudes of members of the American Academy of Orthopaedic Surgeons regarding the treatment of anterior cruciate ligament injury. Arthroscopy 2003; 19(7): 762–70. 12966385 10.1016/s0749-8063(03)00398-0

[CIT0025] Mok Y R, Wong K L, Panjwani T, Chan C X, Toh S J, Krishna L Anterior cruciate ligament reconstruction performed within 12 months of the index injury is associated with a lower rate of medial meniscus tears. Knee Surgery, Sport Traumatol Arthrosc 2019; 27(1): 117–23. 10.1007/s00167-018-5027-y 29978305

[CIT0026] Nordenvall R, Bahmanyar S, Adami J, Mattila V M, Felländer-Tsai L Cruciate ligament reconstruction and risk of knee osteoarthritis: the association between cruciate ligament injury and post-traumatic osteoarthritis: a population based nationwide study in Sweden, 1987–2009. PLoS One 2014; 9(8): e104681. 25148530 10.1371/journal.pone.0104681 PMC4141753

[CIT0027] Parry E, Ogollah R, Peat G Significant pain variability in persons with, or at high risk of, knee osteoarthritis: preliminary investigation based on secondary analysis of cohort data. BMC Musculoskelet Disord 2017; 18(1): 1–11. 28196504 10.1186/s12891-017-1434-3 PMC5310083

[CIT0028] Raynauld J-P, Martel-Pelletier J, Haraoui B, Choquette D, Dorais M, Wildi L M, Abram F, Pelletier J-P Risk factors predictive of joint replacement in a 2-year multicentre clinical trial in knee osteoarthritis using MRI: results from over 6 years of observation. Ann Rheum Dis 2011; 70(8): 1382–8. 21551506 10.1136/ard.2010.146407

[CIT0029] Suter L G, Smith S R, Katz J N, Englund M, Hunter D J, Frobell R, Losina E Projecting lifetime risk of symptomatic knee osteoarthritis and total knee replacement in individuals sustaining a complete anterior cruciate ligament tear in early adulthood. Arthritis Care Res (Hoboken) 2016; 69(2): 201–8. 27214559 10.1002/acr.22940 PMC5121085

[CIT0030] Webster K E, Feller J A, Kimp A, Devitt B M Medial meniscal and chondral pathology at the time of revision anterior cruciate ligament reconstruction results in inferior mid-term patient-reported outcomes. Knee Surgery, Sport Traumatol Arthrosc 2018; 26(4): 1–6. 10.1007/s00167-018-4880-z 29516122

[CIT0031] Weston-Simons J S, Pandit H, Jenkins C, Jackson W F M, Price A J, Gill H S, Dodd C A F, Murray D W Outcome of combined unicompartmental knee replacement and combined or sequential anterior cruciate ligament reconstruction: a study of 52 cases with mean follow-up of five years. Bone Joint J 2012; 94-B(9): 1216–20. 10.1302/0301-620X.94B9.28881 22933493

[CIT0032] Wright F L, Green J, Canoy D, Cairns B J, Balkwill A, Beral V Vascular disease in women: comparison of diagnoses in hospital episode statistics and general practice records in England. BMC Med Res Methodol 2012; 12(1): 161. 23110714 10.1186/1471-2288-12-161 PMC3514155

