# Supplementary material for: Rates of knee arthroplasty in anterior cruciate ligament reconstructed patients: a longitudinal cohort study of 111,212 procedures over 20 years
Source: Acta Orthop. 2019 Jul 10;90(6):568–74. doi: 10.1080/17453674.2019.1639360 (PMC6844427; doi:10.1080/17453674.2019.1639360)
Supplement: Supplemental Material [file IORT_A_1639360_SM5203.pdf]

Supplementary data

Table 1. Demographics of ACLr cohort and proportion of patients with at least 5, 10, and 15 years' follow-up undergoing subsequent TKA

Factor	All index procedures n (%)	5-year outcome n	n TKA	% (95% CI)	10-year outcome n	n TKA	% (95% CI)	15-year outcome n	n TKA	% (95% CI)
Total	111,212 (100)	46,402	212	0.46 (0.40–0.52)	14,292	139	0.97 (0.82–1.2)	3,726	66	1.8 (1.4–2.3)
Sex										
Male	85,905 (77.2)	36,620	127	0.35 (0.29–0.41)	11,672	91	0.78 (0.63–0.96)	3,118	51	1.6 (1.2–2.2)
Female	25,307 (22.8)	9,782	85	0.87 (0.69–1.07)	2,620	48	1.8 (1.4–2.4)	608	15	2.5 (1.4–4.0)
Age group (years)										
< 20	16,156 (14.5)	–	–	–	–	–	–	–	–	–
20–29	47,901 (43.1)	22,503	6	0.03 (0.01–0.06)	6,414	8	0.12 (0.05–0.25)	1,730	6	0.35 (0.13–0.75)
30–39	28,715 (25.8)	14,846	44	0.30 (0.22–0.40)	5,456	45	0.82 (0.60–1.1)	1,533	33	2.2 (1.5–3.0)
40–49	14,690 (13.2)	7,427	99	1.3 (1.1–1.6)	2,028	54	2.7 (2.0–3.5)	403	22	5.5 (3.5–8.2)
50–59	3,235 (2.9)	1,394	41	2.9 (2.1–4.0)	338	24	7.1 (4.6–10.4)	60	5	8.3 (2.8–18.4)
≥ 60	515 (0.46)	232	22	9.5 (6.0–14.0)	56	8	14.3 (6.4–26.2)	–	–	–
Charlson comorbidity index										
0	100,680 (90.5)	43,005	184	0.43 (0.37–0.49)	13,588	128	0.94 (0.79–1.12)	3,726	66	1.8 (1.4–2.3)
1–15	10,484 (9.4)	3,397	28	0.82 (0.55–1.2)	704	11	1.6 (0.78–2.8)	–	–	–
16–30	47 (0.04)	–	–	–	–	–	–	–	–	–
31–50	1 (0.00)	–	–	–	–	–	–	–	–	–
Index of multiple deprivation (quintiles)										
1 = least deprived	23,158 (20.8)	10,230	35	0.34 (0.24–0.48)	3,196	26	0.81 (0.53–1.2)	909	15	1.65 (0.93–2.71)
2	22,290 (20.0)	9,663	48	0.50 (0.37–0.66)	2,963	36	1.2 (0.85–1.7)	824	13	1.58 (0.84–2.68)
3	22,252 (20.0)	9,447	41	0.43 (0.31–0.59)	2,947	25	0.85 (0.55–1.3)	744	16	2.15 (1.23–3.47)
4	21,434 (19.3)	8,599	41	0.48 (0.34–0.65)	2,647	27	1.02 (0.67–1.5)	675	12	1.78 (0.92–3.08)
5 = most deprived	20,233 (18.2)	7,524	45	0.60 (0.44–0.80)	2,210	24	1.1 (0.70–1.6)	510	10	1.96 (0.94–3.58)
Missing	1,845									
Rurality										
Urban	88,727 (79.8)	36,538	160	0.44 (0.37–0.51)	11,272	111	0.98 (0.81–1.2)	2,945	58	1.97 (1.50–2.54)
Rural	21,604 (19.4)	9,489	50	0.53 (0.39–0.69)	2,898	27	0.93 (0.61–1.4)	743	8	1.08 (0.47–2.11)
Missing	881									
Ethnicity										
White	92,820 (83.5)	42,801	212	0.50 (0.43–0.57)	12,840	139	1.1 (0.91–1.3)	3,213	66	2.1 (1.6–2.6)
Asian	5,353 (4.8)	–	–	–	–	–	–	–	–	–
Black	2,131 (1.9)	–	–	–	–	–	–	–	–	–
Mixed	1,401 (1.3)	–	–	–	–	–	–	–	–	–
Other	1,590 (1.4)	–	–	–	–	–	–	–	–	–
Missing	7,917 (7.1)									
Concurrent procedures										
Isolated ACLr	76,773 (69.0)	35,452	180	0.51 (0.44–0.59)	12,577	130	1.0 (0.86–1.2)	3,726	66	1.8 (1.4–2.3)
Chondral surgery	2,601 (2.3)	840	9	1.1 (0.49–2.0)	–	–	–	–	–	–
Meniscal surgery [a]	31,838 (28.6)	10,110	23	0.23 (0.14–0.34)	1,715	9	0.52 (0.24–0.99)	–	–	–

ACLr = anterior cruciate ligament reconstruction; CI = confidence interval; n TKA = number of total or partial knee arthroplasties.
– = suppressed due to small numbers.
[a] with or without concurrent chondral surgery.

APPENDIX: OPCS procedure code list

Procedure	OPCS 4.2	OPCS 4.3	OPCS 4.4	OPCS 4.5	OPCS 4.6	OPCS 4.7	Description
ACLr [a]	W74.2	W74.2	W74.2	W74.2	W74.2	W74.2	Reconstruction of intra-articular ligament NEC
Other ACL[a, b]	W84.1	W84.1	W84.1	W84.1	W84.1	W84.1	Endoscopic repair of intra-articular ligament
Other ACL[a, b]	W84.2	W84.2	W84.2	W84.2	W84.2	W84.2	Endoscopic reattachment of intra-articular ligament
Other ACL[a, b]	W72.3	W72.3	W72.3	W72.3	W72.3	W72.3	Primary prosthetic replacement of intra-articular ligament
Other ACL [a, b]	W72.4	W72.4	W72.4	W72.4	W72.4	W72.4	Prosthetic replacement of intra-articular ligament NEC
Chondral surgery [a]	W83.3	W83.3	W83.3	W83.3	W83.3	W83.3	Endoscopic shaving of articular cartilage
Chondral surgery [a]	W83.8	W83.8	W83.4	W83.4	W83.4	W83.4	Endoscopic articular abrasion chondroplasty
Chondral surgery [a]	W83.8	W83.8	W83.5	W83.5	W83.5	W83.5	Endoscopic articular thermal chondroplasty
Chondral surgery [a]	W83.8	W83.8	W83.6	W83.6	W83.6	W83.6	Endoscopic excision of articular cartilage NEC
Chondral surgery [a]	W83.8	W83.8	W83.8	W83.8	W83.8	W83.8	Other specified therapeutic endoscopic operations on other articular cartilage
Chondral surgery [a]	W83.8	W83.8	W89.1	W89.1	W89.1	W89.1	Endoscopic chondroplasty NEC
Chondral surgery [a]	W83.1	W83.1	W83.1	W83.1	W83.1	W83.1	Endoscopic drilling of lesion of articular cartilage
Chondral surgery [a]	-	-	W84.5	W84.5	W84.5	W84.5	Endoscopic drilling of epiphysis for repair of articular cartilage
Meniscal surgery	W82.2	W82.2	W82.2	W82.2	W82.2	W82.2	Endoscopic resection of semilunar cartilage NEC
Meniscal surgery	W82.3	W82.3	W82.3	W82.3	W82.3	W82.3	Endoscopic repair of semilunar cartilage
Meniscal surgery	W82.1	W82.1	W82.1	W82.1	W82.1	W82.1	Endoscopic total excision of semilunar cartilage
Arthroplasty [a]	W43.1	W43.1	W43.1	W43.1	W43.1	W43.1	Primary total prosthetic replacement of joint using cement NEC
Arthroplasty [a]	W44.1	W44.1	W44.1	W44.1	W44.1	W44.1	Primary total prosthetic replacement of joint not using cement NEC
Arthroplasty [a]	W44.8	W44.8	W44.8	W44.8	W44.8	W44.8	Other specified total prosthetic replacement of other joint not using cement
Arthroplasty [a]	W44.9	W44.9	W44.9	W44.9	W44.9	W44.9	Unspecified total prosthetic replacement of other joint not using cement
Arthroplasty [a]	W45.1	W45.1	W45.1	W45.1	W45.1	W45.1	Primary total prosthetic replacement of joint NEC
Arthroplasty [a]	W45.8	W45.8	W45.8	W45.8	W45.8	W45.8	Other specified other total prosthetic replacement of other joint
Arthroplasty [a]	W45.9	W45.9	W45.9	W45.9	W45.9	W45.9	Unspecified other total prosthetic replacement of other joint
Arthroplasty [a] using cement	W53.8	W53.8	W53.8	W53.8	W53.8	W53.8	Other specified prosthetic replacement of articulation of other bone not
Arthroplasty	W40.1	W40.1	W40.1	O18.1	O18.1	O18.1	Primary hybrid prosthetic replacement of knee joint using cement
Arthroplasty	W40.8	W40.8	W40.8	O18.8	O18.8	O18.8	Other specified hybrid prosthetic replacement of knee joint using cement
Arthroplasty	W40.9	W40.9	W40.9	O18.9	O18.9	O18.9	Unspecified hybrid prosthetic replacement of knee joint using cement
Arthroplasty	W40.1	W40.1	W40.1	W40.1	W40.1	W40.1	Primary total prosthetic replacement of knee joint using cement
Arthroplasty	W40.8	W40.8	W40.8	W40.8	W40.8	W40.8	Other specified total prosthetic replacement of knee joint using cement
Arthroplasty	W40.9	W40.9	W40.9	W40.9	W40.9	W40.9	Unspecified total prosthetic replacement of knee joint using cement
Arthroplasty	W41.1	W41.1	W41.1	W41.1	W41.1	W41.1	Primary total prosthetic replacement of knee joint not using cement
Arthroplasty	W41.8	W41.8	W41.8	W41.8	W41.8	W41.8	Other specified total prosthetic replacement of knee joint not using cement
Arthroplasty	W41.9	W41.9	W41.9	W41.9	W41.9	W41.9	Unspecified total prosthetic replacement of knee joint not using cement
Arthroplasty	W42.1	W42.1	W42.1	W42.1	W42.1	W42.1	Primary total prosthetic replacement of knee joint NEC
Arthroplasty	W42.8	W42.8	W42.8	W42.8	W42.8	W42.8	Other specified other total prosthetic replacement of knee joint
Arthroplasty	W42.9	W42.9	W42.9	W42.9	W42.9	W42.9	Unspecified other total prosthetic replacement of knee joint
Arthroplasty [a]	W58.1	W58.1	W58.1	W58.1	W58.1	W58.1	Primary resurfacing arthroplasty of joint

[a] Additional matched site-specific (knee) code required (Z846, Z765, Z845, Z844, Z774, or Z787)
[b] excluded
